# Can physical education be used to promote social justice? Conceptualizing a framework and thematic domains

**DOI:** 10.1186/s12939-026-02951-7

**Published:** 2026-07-18

**Authors:** Corinne Lovett, Alex Filamor, Negin Fouladi, James Barry, Joshua Bernstein, Basil H. Aboul-Enein

**Affiliations:** 1 Department of Health Policy & Management, School of Public Health, University of Maryland - College Park, 4200 Valley Dr, College Park, MD 20742 USA; 2 Physical Education Teacher Education, East Stroudsburg University , 200 Prospect Street, East Stroudsburg, PA 18301 USA; 3 Doctor of Education in Health Professions Department, College of Graduate Health Studies, A.T. Still University of Health Sciences, 800 W. Jefferson St, Kirksville, MO 63501 USA; 4 College of Arts & Sciences Health & Society Program, University of Massachusetts Dartmouth, 285 Old Westport Rd, North Dartmouth, MA 02747 USA; 5 Faculty of Public Health and Policy, London School of Hygiene & Tropical Medicine, 15-17 Tavistock Place, London, WC1H 9SH UK

**Keywords:** Physical education, Racism, Social justice, Curriculum

## Abstract

Social justice is a concept that is well-recognized yet difficult to define within the context of school curricula. As societies evolve and adapt, physical education (PE) curricula must reflect these changes. Student populations are becoming increasingly diverse, social justice issues and racism remain a public health issue, and the need to review existing school curriculum systems is warranted. This thematic narrative essay examined contemporary literature to see how PE curriculum may be used to promote social justice. After screening for relevance and applying inclusion criteria, 92 articles were included in the thematic analysis. Contemporary peer-reviewed literature on racism, social justice, and physical education produced five qualitative themes: social justice ambiguity in PE, educator bias, dehumanization and marginalization,, neoliberalism in PE, and body image. The review found that colonialism, dominant White cultural norms, educator bias, and narrow body ideals continue to influence modern PE curricula. These findings do not reflect substantial changes in culture and society where social justice is prevalent or needed. Current and new curricula should expand current practice to address the needs of a growing ethnic population, include social justice as a foundational principle, engage in a holistic approach to health that moves beyond body size, and work to humanize our approach to PE. New research into current PE teaching and curricula is warranted.

## Introduction

Physical education (PE) is uniquely positioned within schools because it involves visible demonstrations of ability, health, gender expression, cultural practices, and social interaction. As a result, PE can either reproduce existing social inequities or serve as a space for inclusion, empowerment, and social transformation [1, 2].

Issues related to diversity and equity in schools are not new. Historically, some educational efforts involving students of color emphasized assimilation into dominant White cultural norms while offering limited opportunities for meaningful social advancement. In PE, this matters because many educational systems and teacher preparation programs have traditionally normalized Eurocentric practices, often positioning cultural differences as deviations from the norm. Culturally sustaining and inclusive approaches challenge these patterns by recognizing, valuing, and sustaining the diverse cultural identities, lived experiences, and movement practices of students and communities. Incorporating multiple cultural perspectives into PE can help create more meaningful, equitable, and developmentally appropriate physical activity opportunities for all students [3–5]. 

This paper focuses primarily on PE within the United States, where public schools serve an increasingly diverse student population. According to the National Center for Education Statistics [6], enrollment trends in U.S. public schools show significant demographic changes, with students of color representing a growing proportion of the school-aged population. These demographic shifts are important in PE because many teachers may come from backgrounds that do not fully reflect the cultural identities and lived experiences of the students they teach. At the same time, the literature informing this review includes broader international perspectives from Brazil [7, 8], Sweden [9], Norway [9], Australia [10], New Zealand [9], Israel [11], and the United Kingdom [12, 13]. These perspectives show that issues of equity, inclusion, representation, and access in PE are global concerns, even though they may appear differently across social, political, and cultural contexts [1, 2, 9].

Building from these demographic and international concerns, social justice within PE should not be viewed only through race. While race remains important, students’ experiences are also shaped by gender, disability, socioeconomic status, culture, language, religion, sexuality, and other identities. These identities interact with systems of power and opportunity, shaping students’ access, belonging, participation, and success in PE [2, 14]. For this reason, culturally humble teaching requires educators to recognize students’ lived experiences and community knowledge rather than viewing differences through a deficit-based lens [4, 15].

The increasing diversity of public schools highlights the need for culturally sustaining and socially responsive approaches to PE and health education. Historically marginalized communities often experience disproportionate health disparities, including higher rates of chronic conditions such as hypertension and diabetes [16, 17]. These disparities are connected to broader social determinants of health, including access to healthcare, nutrition, housing, safe recreation spaces, and opportunities for physical activity. PE can support health and wellbeing, but only when instruction recognizes the social, cultural, and economic contexts that shape students’ lives [4, 14, 17].

Within the United States, the PE teacher workforce has historically been predominantly White and middle class, which may create a cultural disconnect between educators and increasingly diverse student populations [4, 18, 19]. While teacher identity alone does not determine teaching effectiveness, limited diversity within the profession may create a cultural disconnect between educators and increasingly diverse student populations. This disconnect may contribute to unconscious bias, deficit-based perspectives, and curriculum choices that do not fully reflect students’ identities or experiences. As a result, current and future physical educators must be prepared to examine their own assumptions, understand systems of power and privilege, and design instruction that is inclusive, culturally relevant, and responsive to diverse learners [4, 15, 18, 19].

These concerns are also connected to broader debates about the purpose of PE, particularly whether PE should focus primarily on individual health responsibility or on the social and structural conditions that shape students’ opportunities for participation [1, 20].

Two competing theoretical perspectives often shape conversations about health and citizenship within PE: neoliberalism and critical social justice pedagogy. Neoliberal approaches tend to emphasize individual responsibility for health and wellness, suggesting that healthy individuals contribute to a healthier society. Critics argue that this view may overlook structural inequities and social determinants of health that influence students’ opportunities for physical activity, wellbeing, and access to resources. Within neoliberal models, PE often emphasizes individual performance, competition, fitness testing, self-sufficiency, and personal responsibility, while broader social and community factors receive less attention [1, 20].

In contrast, social justice-oriented PE emphasizes democratic learning environments, shared power, student voice, culturally responsive teaching, and meaningful participation. In these approaches, teachers serve not only as instructors, but also as advocates, facilitators, and role models who adapt instruction to local cultural and community contexts. This allows students’ lived experiences and identities to shape the learning process. Social justice-oriented PE also asks teachers to challenge traditional assumptions about movement, health, ability, competition, and success [1, 2, 7].

Developing socially just PE requires ongoing critical self-reflection, intentional instruction, and a willingness to question traditional practices. Although many physical educators seek to create positive learning experiences, socially just and culturally sustaining practices have not been consistently implemented in PE programs. Research also suggests that deeply rooted beliefs about teaching and PE can be difficult to change [21, 22]. Even when preservice teachers receive instruction and field experiences related to social justice, they may struggle to apply these practices once they enter school settings [21–23]. Institutional structures within schools may also resist socially critical approaches by reinforcing traditional hierarchies, dominant norms, and established power structures [15, 21–23].

This review is guided by an integrated critical social justice framework that brings together intersectionality, culturally sustaining pedagogy, cultural humility, and critical pedagogy. Intersectionality helps explain how students’ experiences in PE are shaped by overlapping systems of power, including race, gender, disability, social class, culture, language, religion, and access to resources [1, 2, 14]. Culturally sustaining pedagogy extends this work by encouraging PE teachers to recognize, value, and sustain the cultural knowledge, movement practices, and lived experiences students bring into the learning environment [3, 24]. Cultural humility adds an ongoing reflective stance, requiring teachers to examine their own assumptions, biases, and positionality rather than viewing students’ differences through a deficit lens [4, 15]. Critical pedagogy provides the instructional foundation for action by encouraging teachers and students to question traditional power structures, challenge inequitable practices, and create more democratic learning environments [1, 21, 25]. Together, these perspectives frame socially just PE as more than inclusive activity selection; they position curriculum, instruction, assessment, relationships, and teacher preparation as tools for challenging inequity and promoting belonging, voice, and meaningful participation for all students [2, 15, 26, 27].

Using this framework, the purpose of this thematic narrative essay is to propose a conceptual framework and examine thematic domains from contemporary literature related to how PE curricula and instruction may be used to promote social justice across diverse educational contexts.

## Methods

This study employed a thematic narrative review to examine contemporary literature related to social justice, equity, inclusion, race, culture, teacher bias, body image, and critical pedagogy within PE and PETE. A thematic narrative review was selected because the purpose of the study was not to determine intervention effectiveness but rather to identify recurring conceptual patterns and theoretical perspectives across diverse bodies of literature [28].

Searches were conducted using PubMed, Scopus, SPORTDiscus, ERIC, and ScienceDirect. To supplement these searches and ensure comprehensive coverage of conceptual and interdisciplinary scholarship, publisher platforms including SAGE Journals Online, Taylor & Francis Online, Wiley Online Library, and SpringerLink were also searched because they host many of the leading journals in physical education, sport pedagogy, curriculum studies, and teacher education.

Search terms included combinations of keywords and phrases such as social justice physical education, critical pedagogy, culturally responsive teaching, culturally sustaining pedagogy, race and physical education, racism in physical education, equity in PE, inclusion in physical education, teacher bias, body image, neoliberalism, physical education teacher education, and PETE. Boolean operators such as ‘AND’ and ‘OR’ were used to combine search terms and broaden or narrow the search as needed.

Articles were selected based on their relevance to social justice, equity, inclusion, race, culture, teacher bias, body image, critical pedagogy, and PE curriculum or teacher education. The review focused primarily on contemporary literature published between 2013 and 2021, while also including selected foundational works that were important to the development of social justice, culturally sustaining pedagogy, and critical pedagogy. Literature from multiple international contexts was included to provide a broader understanding of social justice in PE across different educational and cultural settings.

Only peer-reviewed journal articles, conceptual papers, empirical studies, and scholarly reviews published in English were included. Articles were excluded if they did not directly address PE, health and PE, PETE, school-based physical activity, social justice, equity, inclusion, or related sociocultural issues.

Articles were not required to address all identified concepts simultaneously. Rather, studies were included if they focused on physical education, physical education teacher education, health and physical education, or school-based physical activity and addressed at least one social justice-related construct relevant to the review. These constructs included equity, inclusion, race and racism, culture, disability, marginalization, teacher bias, critical pedagogy, culturally responsive or culturally sustaining practices, body image, neoliberalism, or related sociocultural issues. Because social justice is a multidimensional construct, many articles addressed different yet overlapping aspects of equity, inclusion, and educational justice. Following article selection, studies were coded according to the primary and secondary concepts addressed within each article and subsequently organized into broader thematic categories that reflected recurring patterns across the literature.

Literature from multiple international contexts, including the United States, Brazil, Sweden, Norway, Australia, New Zealand, Israel, and the United Kingdom, was included to provide broader cultural and educational perspectives. Incorporating studies from diverse national settings allowed for examination of both shared and context-specific challenges related to social justice and equity in PE. Although only English-language publications were included, the international scope of the literature provided valuable insight into how social justice is understood and enacted across different educational systems and sociocultural environments.

Data from the selected articles were organized in an article tracking matrix in Microsoft Excel. The matrix included author(s), year, title, research design, focus area, key findings, initial codes, and emerging themes. This process allowed the researchers to compare findings across studies and identify recurring concepts.

Following article selection, the literature was read and independently reviewed and coded by CL, AF, NF, JBa, and BHA-E. The researchers subsequently met to compare codes, discuss discrepancies, and establish consensus. Disagreements were resolved through discussion and majority agreement. During the initial review, articles were examined for recurring concepts, tensions, perspectives, and issues related to social justice within PE and PETE. Open coding was used to identify frequently occurring ideas and patterns across studies [28, 29], allowing the research team to remain close to the data before organizing codes into broader categories and themes. During later reviews, similar codes were compared, refined, and grouped into broader conceptual categories through discussion among the research team. When reviewers differed in their interpretation of article relevance, coding, or theme placement, disagreements were discussed by the research team and resolved through majority agreement [30]. During this process, several related categories were identified, including critical pedagogy, activist pedagogy, inclusion, disability, race, culture, decolonizing curriculum, social justice practice, teacher learning, and body surveillance. For clarity in reporting, these categories were collapsed into five overarching themes: neoliberalism in PE, social justice ambiguity in physical education, educator bias, dehumanization and marginalization, and body image [28–30].

For clarity in reporting, these categories were collapsed into five overarching themes: neoliberalism in PE, social justice ambiguity in physical education, educator bias, dehumanization and marginalization, and body image. Because individual articles often addressed multiple social justice-related concepts, studies were permitted to contribute to more than one thematic category. The themes therefore represent broader conceptual domains rather than mutually exclusive classifications of the literature. The final stage of analysis involved synthesizing related categories into overarching themes that captured the dominant patterns within the literature.

### Positionality and reflexivity

The authors recognize that their own professional backgrounds, cultural experiences, disciplinary training, and social positions may have influenced how the literature was interpreted. Because this review addresses racism, equity, inclusion, and social justice, the research team engaged in collaborative discussion throughout the coding and theme development process to reduce the likelihood that findings reflected only one author’s perspective. While this process strengthened the trustworthiness of the analysis, the authors acknowledge that all thematic interpretation is shaped by researcher positionality and should be understood as situated rather than neutral.

## Results

After screening for relevance and applying the inclusion criteria, 92 articles met the criteria and were included in the thematic analysis. Articles were reviewed for recurring concepts related to social justice, equity, inclusion, race, culture, teacher bias, dehumanization, body image, and neoliberalism within PE and PETE.

Five major themes were identified: Social Justice Ambiguity in PE, Educator Bias, Dehumanization and Marginalization, Neoliberalism and PE, and Body Image. Educator Bias appeared most frequently, with 68 coded occurrences. This theme included teacher assumptions, whiteness, deficit thinking, cultural responsiveness, anti-racist practice, privilege, and teacher preparation. Dehumanization and Marginalization appeared in 57 coded occurrences and reflected concerns related to exclusion, othering, bullying, lack of belonging, disability marginalization, and reduced access to meaningful participation.

Social Justice Ambiguity appeared in 55 coded occurrences, Articles within this theme frequently discussed social justice as a guiding principle for physical education but differed substantially in how the concept was defined and operationalized. Common subthemes included equity, inclusion, democratic participation, student voice, advocacy, and culturally responsive teaching. While many authors supported socially just practices, there was limited consensus regarding what social justice should look like in practice within PE settings.

Educator Bias was the most frequently occurring theme, with 68 coded occurrences. This theme focused on how teacher beliefs, assumptions, cultural backgrounds, and professional preparation may influence instructional decisions and student experiences. Subthemes included whiteness, privilege, deficit thinking, anti-racist practice, cultural responsiveness, and teacher education. Many studies highlighted the need for greater critical reflection and preparation related to diversity, equity, and inclusion.

Dehumanization appeared in 57 coded occurrences. Articles within this theme described experiences of exclusion, othering, stereotyping, and unequal participation opportunities. Subthemes included disability marginalization, racial and cultural exclusion, bullying, social isolation, and barriers to meaningful engagement in physical education. Several studies also examined the influence of colonialism and dominant cultural norms on curriculum and instructional practices.

Neoliberalism and Physical Education appeared in 21 coded occurrences. This theme reflected literature examining the emphasis placed on individual responsibility, competition, accountability, fitness testing, and performance outcomes. The reviewed articles frequently discussed how health and success were framed as individual achievements while broader social and structural influences received less attention. Subthemes included fitness surveillance, personal responsibility, performance measurement, and accountability.

Body Image was identified in 10 coded occurrences. Articles in this theme examined how physical education may contribute to body surveillance, appearance-based judgments, obesity discourse, and narrow definitions of health and fitness. Subthemes included gendered body expectations, idealized body norms, weight-related stigma, and the relationship between body image and participation in physical activity.

**Fig. 1:**
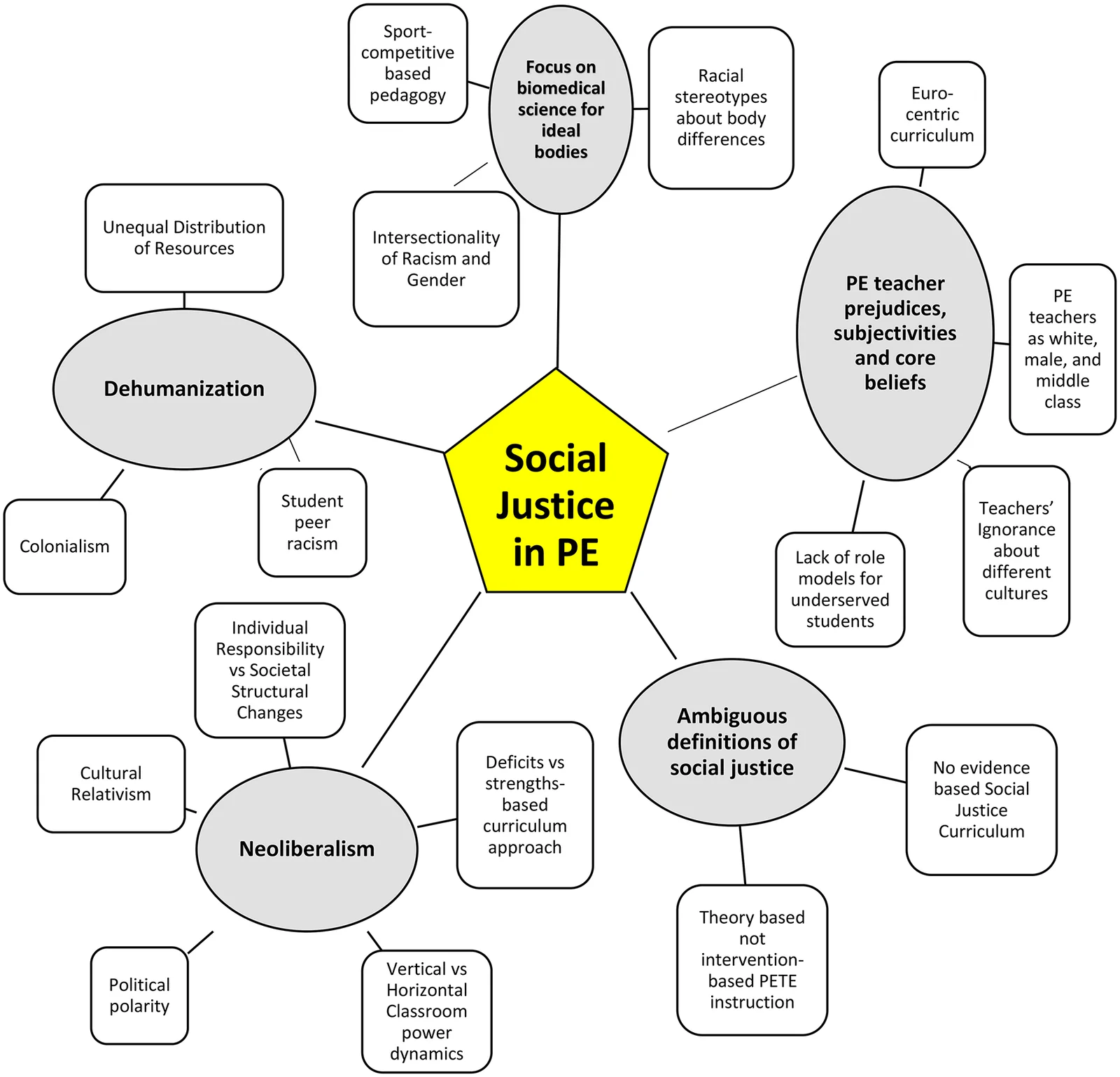
Conceptual framework and thematic domains

Several additional subthemes were identified across the literature and are summarized in Table 1. A conceptual framework illustrating the relationships among the major themes and domains is presented in Fig. 1.

**Table 1:** Major themes and theme occurrences across the literature

Main Theme	Supporting Subthemes/Categories	Theme Occurrences Across Articles	Core Focus
Social Justice Ambiguity in PE	Social justice ambiguity, critical pedagogy, activist pedagogy, democratic teaching, student voice, advocacy, equity versus equality, and social justice practice	55	How social justice is defined, interpreted, and applied differently across PE and PETE contexts?
Educator Bias	Teacher assumptions, implicit bias, whiteness, race, culture, privilege, deficit thinking, anti-racism, culturally responsive teaching, decolonizing curriculum, and teacher preparation	68	How teacher beliefs, assumptions, and preparation shape equity, inclusion, and student experiences in PE and PETE?
Dehumanization and Marginalization	Inclusion, access, disability, exclusion, bullying, misrecognition, lack of belonging, limited accommodations, and stereotyping	57	How students are marginalized through exclusionary practices, limited access, reduced belonging, and lack of meaningful support?
Neoliberalism and Physical Education	Market-driven PE, privatization, accountability, individual responsibility, fitness testing, health surveillance, competition, and performance-based practices	21	How PE can emphasize personal responsibility, competition, performance, surveillance, and market-based approaches to health and education?
Body Image	Body image, gender norms, body surveillance, obesity discourse, appearance standards, health-based body ideals, masculinity, femininity, and fitness norms	10	How narrow definitions of health, fitness, body size, appearance, and performance influence student experiences in PE?

Note. Theme counts represent coded theme occurrences across the reviewed articles. Articles could be coded under more than one theme, so counts are not mutually exclusive and should not be added together

## Discussion

Collectively, the findings suggest that PE is not a neutral educational space. Decisions regarding curriculum, assessment, instructional practices, and definitions of success are shaped by broader social, cultural, and political influences that can either challenge or reproduce educational inequities. Across the reviewed studies, neoliberal ideas, deficit-based perspectives, and narrow definitions of health and ability were commonly identified as barriers to socially just PE practices [1, 20]. At the same time, the literature highlighted teaching approaches that may help create more inclusive and humanizing PE experiences. Researchers emphasized the importance of student voice, culturally responsive teaching, collaboration, critical reflection, and community engagement as ways to support more equitable PE environments [1–3]. The findings suggested that socially just PE should move beyond only teaching sports and fitness skills and instead help students develop confidence, belonging, and positive lifelong relationships with physical activity.

### Ambiguity of social justice in physical education

The findings from this review suggest that social justice remains a complex and often inconsistently defined concept within PE. Although most researchers agreed that social justice is concerned with creating equitable opportunities for all students, the literature revealed considerable variation in how the concept was understood and applied [1, 26]. Some studies emphasized fairness and equal access to learning opportunities, while others focused on inclusion, student voice, culturally responsive teaching, advocacy, and addressing inequities related to race, gender, disability, and social class [1, 2]. This lack of a shared definition emerged as an important finding because it may contribute to inconsistent implementation of social justice practices across schools, teacher education programs, and PE classrooms [1]. If educators hold different understandings of social justice, they may also differ in how they design curriculum, assess students, and create inclusive learning environments.

Social justice is often broadly defined as the equitable distribution of opportunities, resources, and participation while actively addressing systems that create disadvantages and exclusions [38]. Within PE, however, researchers have applied the concept in different ways, contributing to ambiguity regarding its implementation in practice. The literature also suggested that social justice in PE extends beyond curriculum content and is closely connected to classroom relationships, power structures, and student experiences. Researchers found that democratic teaching approaches and shared decision-making opportunities can help create more inclusive learning environments by increasing student voice, participation, and feelings of belonging [2, 25]. When students are encouraged to contribute ideas, make choices, and take ownership of aspects of their learning, they may be more likely to view themselves as valued members of the learning community. These findings suggest that socially just PE is not simply about what is taught, but also about how teaching and learning occur [2].

Another important finding was the tension between traditional PE practices and socially just approaches to teaching. Many PE programs continue to emphasize competition, fitness testing, grading, and sport performance as measures of student success [1, 20]. While these practices have long been embedded within PE, researchers suggested that they may conflict with goals related to inclusion, equity, and meaningful participation for all students [1, 2]. The issue is not the inclusion of competition or sport within PE, but rather when these elements become the dominant measures of achievement and participation. Students who have access to club sports, private coaching, transportation, and athletic opportunities outside of school often enter PE with experiences and advantages that other students may not possess. Consequently, success may be interpreted as individual effort or ability while overlooking broader social and economic factors that influence participation opportunities [14].

From a social justice perspective, these findings highlight the importance of broadening how success is defined within PE. Researchers argued that socially just PE should value effort, improvement, cooperation, leadership, and meaningful participation alongside skill development and physical performance [1, 2, 26, 27]. Such an approach recognizes that students enter PE with diverse backgrounds, experiences, abilities, and opportunities. When PE programs focus too narrowly on competition and performance outcomes, they may unintentionally reinforce inequities and discourage some students from developing positive relationships with movement and physical activity [20]. Conversely, learning environments that emphasize inclusion, cultural responsiveness, student voice, and physical literacy may better support meaningful participation and lifelong engagement in physical activity for all students [2, 3].

### Educator biases and perspectives

Educator biases, perspectives, and lived experiences emerged as a major theme in the literature. Both implicit and explicit biases can influence instructional decisions, student interactions, expectations, grouping practices, and perceptions of student ability [15]. These biases are often shaped by personal experiences, teacher preparation, culture, social class, gender, race, and broader educational systems [4, 31]. The literature also highlighted that the PE teaching workforce in the United States remains predominantly White and middle class, which may create a cultural disconnect between educators and increasingly diverse student populations [4, 18, 19]. While teacher identity alone does not determine teaching effectiveness, limited diversity within the profession may reduce opportunities for students to see their racial, cultural, linguistic, and socioeconomic experiences reflected in PE settings. Therefore, increasing diversity in the PE teaching workforce should be paired with teacher preparation that explicitly addresses equity, cultural responsiveness, anti-racism, and critical self-reflection [4, 15, 32].

PE curricula and teacher preparation programs may also reproduce Eurocentric traditions and Western views of sport, competition, and movement [7, 31]. When these traditions are presented as neutral or universal, movement practices outside dominant traditions may receive less attention [7]. Teachers who feel unprepared to discuss race, culture, privilege, and social justice may unintentionally allow stereotypes and inequities to continue [15, 31].

Student experiences in PE are shaped by intersecting social factors, including race, social class, gender, disability, culture, language, and access to resources [2, 14]. Students with access to transportation, organized sports, private coaching, safe recreation spaces, and financial resources may enter PE with advantages unavailable to many of their peers [14, 33]. As a result, confidence, skill development, and participation may reflect differences in opportunity rather than differences in motivation or ability [2, 14]. Sato et al. [34] illustrated this point through the swimming experiences of African American preservice PE teachers. Many participants reported limited swimming experience because of historical inequities in aquatic access and negative experiences in swimming spaces. As participants received instruction and support, their confidence and competence increased, challenging assumptions about innate ability and showing how access and opportunity shape physical activity participation [34].

Ultimately, physical education has the potential to support not only lifelong physical activity but also empathy, social awareness, critical thinking, and respect for diversity [2, 25]. Anti-racist and socially just teaching extends beyond skill instruction and sport participation by recognizing how broader social conditions influence health, opportunity, participation, and wellbeing. Bias becomes problematic not because educators intentionally seek to exclude students, but because unconscious assumptions may shape expectations, opportunities, and experiences within PE environments. Recognizing and addressing these assumptions is an important step toward creating more equitable and inclusive PE experiences for all students.

### Dehumanization and marginalization

Dehumanization and marginalization emerged as recurring concerns in the literature [2, 35]. In PE, dehumanization can occur when students are reduced to stereotypes, physical traits, perceived ability, race, culture, gender, disability, or athletic performance rather than being recognized as whole people with diverse strengths, histories, and experiences [14, 36]. These assumptions are shaped by colonialism, racism, unequal power structures, and dominant sport traditions that continue to influence PE curriculum and instruction [7, 35, 36].

Racial and social stereotypes can also shape expectations within PE and sport settings [14, 37]. Students may be encouraged toward certain activities because of assumptions connected to race, gender, social class, or perceived athletic ability rather than their personal interests, goals, or experiences [14, 33]. Although teachers may not intend to reinforce stereotypes, implicit biases embedded in curriculum choices, sport traditions, grouping practices, and expectations can limit students’ opportunities for meaningful participation and belonging [2, 37].

Studies from Brazil and New Zealand further show how colonial histories continue to shape PE curriculum and teacher education [7, 8, 35]. Research in Brazil described PE curriculum traditions as influenced by European, elitist, heteronormative, and adult-centered perspectives, with discussions of racism and colonialism often minimized or avoided [7, 8]. In New Zealand, efforts to include Māori games, histories, and cultural perspectives were sometimes resisted by preservice teachers who viewed these practices as unnecessary or outside traditional PE [35]. These findings suggest that colonial assumptions remain embedded in some PE structures and influence how culture, identity, and knowledge are valued [7, 8, 35].

Together, these findings suggest that socially just PE requires more than avoiding individual prejudice. It requires teachers and teacher education programs to examine curriculum, instructional practices, school norms, and assumptions about students. Building relationships, valuing diverse cultural experiences, and creating equitable opportunities for participation can help challenge dehumanizing practices and support more inclusive PE environments [2, 25].

### Ambiguity of social justice in PE

The findings from this review suggest that social justice remains a complex and often inconsistently defined concept within PE. Although most researchers agreed that social justice is concerned with creating equitable opportunities for all students, the literature revealed considerable variation in how the concept was understood and applied [1, 26]. Some studies emphasized fairness and equal access to learning opportunities, while others focused on inclusion, student voice, culturally responsive teaching, advocacy, and addressing inequities related to race, gender, disability, and social class [1, 2]. This lack of a shared definition emerged as an important finding because it may contribute to inconsistent implementation of social justice practices across schools, teacher education programs, and PE classrooms [1]. If educators hold different understandings of social justice, they may also differ in how they design curriculum, assess students, and create inclusive learning environments.

Social justice is often broadly defined as the equitable distribution of opportunities, resources, and participation while actively addressing systems that create disadvantages and exclusions [38]. Within PE, however, researchers have applied the concept in different ways, contributing to ambiguity regarding its implementation in practice. The literature also suggested that social justice in PE extends beyond curriculum content and is closely connected to classroom relationships, power structures, and student experiences. Researchers found that democratic teaching approaches and shared decision-making opportunities can help create more inclusive learning environments by increasing student voice, participation, and feelings of belonging [2, 25]. When students are encouraged to contribute ideas, make choices, and take ownership of aspects of their learning, they may be more likely to view themselves as valued members of the learning community. These findings suggest that socially just PE is not simply about what is taught, but also about how teaching and learning occur [2].

Another important finding was the tension between traditional PE practices and socially just approaches to teaching. Many PE programs continue to emphasize competition, fitness testing, grading, and sport performance as measures of student success [1, 20]. While these practices have long been embedded within PE, researchers suggested that they may conflict with goals related to inclusion, equity, and meaningful participation for all students [1, 2]. The issue is not the inclusion of competition or sport within PE, but rather when these elements become the dominant measures of achievement and participation. Students who have access to club sports, private coaching, transportation, and athletic opportunities outside of school often enter PE with experiences and advantages that other students may not possess. Consequently, success may be interpreted as individual effort or ability while overlooking broader social and economic factors that influence participation opportunities [14].

From a social justice perspective, these findings highlight the importance of broadening how success is defined within PE. Researchers argued that socially just PE should value effort, improvement, cooperation, leadership, and meaningful participation alongside skill development and physical performance [1, 2, 26, 27]. Such an approach recognizes that students enter PE with diverse backgrounds, experiences, abilities, and opportunities. When PE programs focus too narrowly on competition and performance outcomes, they may unintentionally reinforce inequities and discourage some students from developing positive relationships with movement and physical activity [20]. Conversely, learning environments that emphasize inclusion, cultural responsiveness, student voice, and physical literacy may better support meaningful participation and lifelong engagement in physical activity for all students [2, 3].

### Neoliberalism in PE

Neoliberalism still shapes how many PE programs are taught and assessed. Across the literature, neoliberal ideas were connected to individual responsibility, competition, accountability, performance outcomes, fitness testing, body monitoring, and standardized measures of health and fitness [1, 20]. These practices often position success as a matter of personal effort while overlooking structural barriers such as poverty, racism, disability, transportation, neighborhood safety, and access to organized sport [14, 33, 39]. The concern is not the inclusion of sport or fitness education, but rather when these elements become the main measures of achievement, participation, and student success [1, 2].

One way to push back against this model is to give students more voice in PE. Democratic and student-centered approaches can challenge traditional power structures by allowing students to participate in decision-making and shape parts of the learning process [2, 40]. When teachers use shared power structures, students may be more likely to participate and remain engaged in class activities [40]. Students may also feel more included and valued when PE teachers create learning environments that encourage teamwork, collaboration, student voice, and shared responsibility [2].

PE curriculum should also move beyond traditional Western sports and activities [8, 10]. Many PE programs focus mainly on sports such as basketball, football, soccer, and volleyball, which are often connected to Western traditions [10]. Researchers found that including Indigenous games and culturally diverse movement activities may help students feel more represented and included in PE classes while also challenging narrow views of what counts as meaningful physical activity [8, 10].

Buroinjin offers one example of how PE can include different movement traditions. Buroinjin is a traditional Aboriginal Australian game that has been used to challenge sport-centered PE curriculum and introduce students to different ways of learning through movement and play [10]. However, Indigenous games and culturally diverse movement practices should be taught carefully to avoid tokenism or cultural appropriation [3, 4, 8]. Teachers should present these activities with attention to their historical, cultural, and community meanings rather than treating them as isolated “new games” [8, 10]. When possible, educators should consult community members, use accurate cultural sources, acknowledge the origins of activities, and avoid altering practices in ways that erase their meaning [3, 4].

Teachers can also use reflective approaches, such as self-study and Teaching Games for Understanding, to make PE more inclusive [10]. Rather than focusing only on competition and skill performance, these approaches encourage students to think critically, work together, solve problems, and understand the purpose behind games and movement activities [10]. Overall, PE programs that emphasize inclusion, student voice, cultural responsiveness, meaningful participation, and physical literacy may help students build confidence, feel valued, and develop positive relationships with lifelong physical activity [1, 2].

### Body image

Body image emerged as an important theme because PE can unintentionally reinforce narrow definitions of health, fitness, and appearance [11, 20]. Fitness scores, body measurements, performance standards, and ideas about the “ideal” body may shift attention toward evaluating students’ bodies rather than helping students build positive relationships with movement [20]. From a social justice perspective, the concern is not promoting health but defining health through limited measures that may privilege certain body types, abilities, and experiences while marginalizing others [14, 20]. These pressures may be especially harmful for students whose bodies, abilities, cultural backgrounds, or access to resources do not match dominant expectations of fitness or athletic success [11, 14].

These findings suggest that PE teachers should broaden how health and success are defined in their programs. Rather than focusing mainly on appearance, body size, or performance outcomes, socially just PE should promote holistic wellbeing, physical literacy, personal growth, and meaningful participation [2, 41]. This approach recognizes that students enter PE with different bodies, abilities, identities, and experiences [2, 14]. By valuing effort, improvement, enjoyment, inclusion, and student voice, PE teachers can help students develop more positive relationships with movement while reducing stigma, exclusion, and negative body image experiences [2, 20].

### Implications for physical education teachers

PE teachers play an important role in creating inclusive and equitable learning environments [1, 2]. Social justice in PE requires teachers to examine how their beliefs, instructional practices, assessments, and classroom environments shape student experiences [15, 25]. Ongoing reflection and professional learning related to culture, equity, bias, disability, and inclusion can help teachers better support diverse learners [4, 15]. Cultural humility is especially important because it encourages teachers to recognize students’ lived experiences and community knowledge rather than viewing differences through a deficit-based lens [4].

Teachers can support socially just PE by offering student voice and choice, building meaningful relationships, using culturally responsive practices, and creating multiple pathways for success [1, 2]. This means valuing effort, improvement, cooperation, leadership, skill development, and meaningful participation rather than relying only on competition, fitness testing, or athletic performance [14, 20]. These practices can help students experience PE as a place of belonging, participation, and personal growth rather than a space that rewards only the most skilled or most experienced students [2, 25].

PETE programs also play an important role in preparing future teachers [4, 15]. Preservice teachers need coursework and field experiences that address social justice, culturally responsive teaching, disability inclusion, race, privilege, identity, and critical reflection [7, 32, 39]. These experiences can help future teachers recognize and challenge assumptions that contribute to inequitable PE experiences [15, 25]. Teacher preparation programs should also provide opportunities for candidates to work with students from diverse racial, cultural, linguistic, socioeconomic, and ability backgrounds so that future teachers are better prepared to create inclusive PE environments [4, 7, 39].

Although individual teachers play an important role in creating socially just PE environments, meaningful change also requires attention to institutional and structural barriers that shape what teachers can do in practice [1, 2]. Teachers may want to use culturally responsive, student-centered, and inclusive practices, but their choices are often shaped by school policies, scheduling constraints, limited funding, large class sizes, assessment mandates, facilities, curriculum requirements, and access to professional development [15, 20]. For example, when PE programs are evaluated primarily through fitness testing, sport performance, or standardized accountability measures, teachers may feel pressure to prioritize measurable outcomes over student voice, belonging, cultural relevance, and meaningful participation [1, 20]. Similarly, limited resources, inequitable access to safe facilities, and narrow curricular expectations can make it difficult for teachers to include diverse movement practices or adapt instruction for students with different abilities, identities, and experiences [2, 10, 39]. Socially-just PE therefore requires support from school leaders, districts, teacher education programs, and professional organizations [2, 15]. These groups can help by funding inclusive equipment, supporting smaller and safer class structures, providing ongoing professional development, recruiting and retaining a more diverse PE teacher workforce, and adopting assessment systems that value effort, growth, cooperation, leadership, and meaningful participation alongside skill development and physical literacy [2, 15, 19, 26].

### Limitations

This review has several limitations. First, only English-language publications were included, which may have excluded important scholarship published in other languages. Second, because this was a thematic narrative review rather than a systematic review or meta-analysis, the findings should be interpreted as conceptual patterns rather than evidence of intervention effectiveness. Third, although the review included international literature, the discussion remains influenced by scholarship from the United States and other English-language contexts.

A further limitation is that social justice encompasses a broad range of interconnected concepts, including race, culture, disability, inclusion, equity, and critical pedagogy. Consequently, articles addressing different dimensions of social justice were included in the review. While this approach allowed for a more comprehensive examination of the literature, some thematic categories contain overlapping concepts that may be interpreted differently across educational and cultural contexts.

## Conclusion

Socially-just PE should not be understood only as the responsibility of individual teachers. It also requires institutional commitment, supportive policies, equitable resources, inclusive teacher preparation, and assessment systems that recognize the diverse ways students experience, access, and benefit from physical education.

## Data Availability

No datasets were generated or analysed during the current study.
